# Detection of a white cataract with elevated intralenticular pressure using an optical biometer based on swept-source optical coherence tomography

**DOI:** 10.1007/s10384-025-01284-2

**Published:** 2025-09-26

**Authors:** Shota Kamei, Kouhei Hashizume, Junya Kizawa, Daijiro Kurosaka

**Affiliations:** Department of Ophthalmology, School of Medicine, Iwate Medical University, 2-1-1 Idaitori, Shiwa-gun, Iwate, Japan

**Keywords:** White cataract, SS-OCT, Biometer, Lens thickness, Anterior chamber depth

## Abstract

**Purpose:**

To determine whether a white cataract (WC) with elevated intralenticular pressure (EWC) can be distinguished from one with normal intralenticular pressure (NWC) using an optical biometer based on Swept-Source Optical Coherence Tomography (SS-OCT).

**Study design:**

Retrospective observational study

**Methods:**

Patients with a WC who had undergone phacoemulsification and intraocular lens (IOL) implantation at Iwate Medical University Hospital were enrolled. Using surgical videos, WCs with/without leakage of the liquefied cortex or bulging of the swollen cortex were classified as EWC and NWC, respectively. Both groups were compared in terms of age, sex, ocular comorbidities, various parameters, and the presence of characteristic appearances of the liquefied cortex on SS-OCT images. If the parameters were not measured appropriately, remeasurement using SS-OCT images were performed.

**Results:**

Of the 48 patients with a WC, 26 eyes were classified as having an EWC, and 22 as NWC. There were significant differences in lens thickness (LT), anterior chamber depth (ACD), ΔLT, ΔACD (the differences in LT or ACD between the eyes with a WC and the fellow eyes, respectively), and age between an EWC and an NWC. ROC analysis showed that the AUC values of LT, ΔLT, and ΔACD were higher than 0.970. LT required remeasurement in approximately half of the WC, whereas ACD did not. Image evaluation for detecting an EWC had a sensitivity of 88.5% and a specificity of 90.9%.

**Conclusions:**

An optical biometer based on SS-OCT may detect an EWC using LT, ΔLT, ΔACD, and SS-OCT images.

## Introduction

Several types of cataracts are classified as white cataracts (WCs) [1–3]. One of these is caused by the degeneration and liquefication of the cortex, elevating intralenticular pressure (ILP). In patients with elevated ILP WCs (EWCs), surgical problems are likely to occur during continuous curvilinear capsulorhexis (CCC) [1–3]. ILP sometimes causes uncontrollable peripheral extension of the anterior capsular flap, leading to posterior capsule rupture [4, 5]. Various methods are reportedly used to prevent these complications, including anterior capsular dyeing [6], aspiration of the liquefied cortex [7], two-stage capsulorhexis [8, 9]. diathermic high-frequency capsulorhexis [10], and femtosecond laser-assisted capsulorhexis [11]. In contrast, in the type of WC coming from the cortex and the nucleus being white, ILP is not elevated (normal ILP WC; NWC) [1–3]. The nucleus is often hard, but surgery is possible, similar to common cataracts [1, 2]. To choose and use appropriate methods for each type of WC, it is essential to correctly evaluate the characteristics of WCs and detect EWC before surgery [12–15].

Several methods are reported for the detection of EWCs. Because the classification cannot be confirmed by slit-lamp biomicroscopy alone [3], other methods using A-scan ultrasonography [3, 16], anterior-segment optical coherence tomography (AS-OCT) [17], and Scheimpflug tomography [15] are reported. Decisions on which method to use are based on characteristic findings in the images and measurements such as lens thickness (LT) and anterior chamber depth (ACD). Although these methods are useful, the accuracy of A-scan ultrasound measurements is affected by the operator’s experience and the handling of the probe tip [18]. Special equipment or equipment improvements are required to obtain AS-OCT or Scheimpflug images [11]. However, these methods are not widely applied.

Biometry is necessary for the preoperative measurement of the intraocular lens (IOL) power calculation. Optical biometry has many advantages over ultrasound and immersion biometry, being more manageable, faster, and more accurate, and is accepted in almost all countries [19]. Among the various optical biometers, those based on swept-source OCT (SS-OCT) technology are likely to become the gold standard for ocular biometry owing to their excellent repeatability and reproducibility [20]. Optical biometers based on SS-OCT technology measure various parameters, including LT and ACD, and show SS-OCT images as well. However, the classification of WCs using optical biometers based on SS-OCT has not yet been examined. Although these biometric measurements are influenced by factors such as age, gender, and AL, and vary between individuals [21–23], they are usually symmetrical between the left and right eyes of the same individual [22, 24, 25]. To evaluate whether EWC can be detected using measurements and SS-OCT images obtained with an optical biometer based on SS-OCT, and whether comparing left-right differences is of any value, a retrospective observational study was conducted using the IOLMaster 700 (Carl-Zeiss-Meditec).

## Methods

### Study population

This retrospective case series included all consecutive adult patients with WC who underwent phacoemulsification and IOL implantation at the Iwate Medical University Hospital between 1 August 2020 and 31 August 2021. To examine the differences in LT and ACD between the eye with a WC and the fellow eye, cases in which the fellow eye had undergone cataract surgery previously or both eyes had a WC were excluded. We also excluded eyes with a Morgagnian-type WC or previous ocular surgery. The study adhered to the principles of the Declaration of Helsinki and was approved by the Institutional Review Board of Iwate Medical University (MH2021-171). The committee at Iwate Medical University determined that patient informed consent was not necessary to use their medical record data.

### Clinical assessment and data collection

**Fig. 1.**
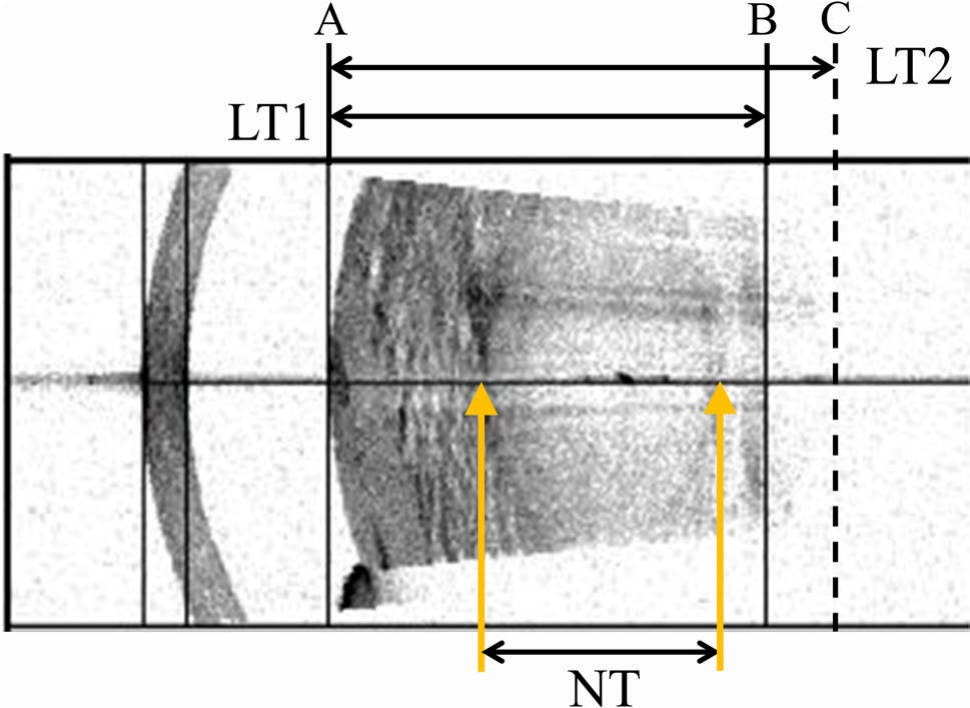
Swept-Source Optical Coherence Tomography (SS-OCT) image of anterior portion. The IOLMaster 700 determined the anterior (line A) and posterior (Line B) surfaces of the lens and displayed the distance between them (lens thickness 1; LT1). However, the posterior surface of the lens had been misjudged. The line of the posterior surface of the lens was reset to an appropriate position (line C). Using the ImageJ software, the distances of LT1 and LT2 on the image were measured to calculate the ratio (LT2/LT1). The true LT (LT2) was the value obtained from multiplying LT1 (as displayed by the IOLMaster) by its ratio. The orange arrows showed the borders between lens nucleus and cortex. In the same way, the distances of LT2 and Nuclear thickness (NT) on the image were measured using the ImageJ software to calculate the NT ratio (NT/LT2). NT was the value obtained from multiplying true LT2 by the NT ratio.

Medical records were analyzed for age at cataract surgery, sex, and ocular comorbidities. We collected the ACD (distance between the anterior surface of the cornea and the anterior surface of the lens), LT, and the axial length (AL) from the IOLMaster. whenever the IOLMaster cannot measure appropriately, it will display a warning or a failed sign. Even with a success sign, which means the measurements were appropriately conducted, the image of SS-OCT was checked. When these data were confirmed as inappropriate, they were remeasured (Fig. 1). In the LT, the inappropriate line of the anterior and/or posterior surface of the lens, which the IOLMaster had determined, was reset to an appropriate position, and the distance was measured using the ImageJ software (National Institutes of Health). The ratio between the newly set distance and the original distance was calculated. The remeasured LT was calculated by multiplying the ratio by the LT shown by IOLMaster. The same procedure was used for the ACD. When the IOLMaster displayed an AL failed notice and we could not determine the fundus edge in the image of SS-OCT with either eye, AL measurement by ultrasound (AL-4000, Tomey) was used instead.

To obtain the cortical thickness (CT) and nuclear thickness (NT) of the lens, we divided the lens into three parts, the anterior cortex, nucleus, and posterior cortex, using the methods reported by Wang et al. [26] Using ImageJ software, the distance of the nuclear part (NT) was measured (Fig. 1). The ratio of NT to LT (NT ratio) was also calculated. The CT was obtained by subtracting NT from LT.

The difference in LT between the eyes with a WC and the fellow eyes (ΔLT) was calculated by subtracting the LT of the fellow eye from the LT of the eye with a WC. ΔACD was also calculated using the ACD of the eyes with a WC and the fellow eyes.

### Classification of WCs

**Fig. 2.**
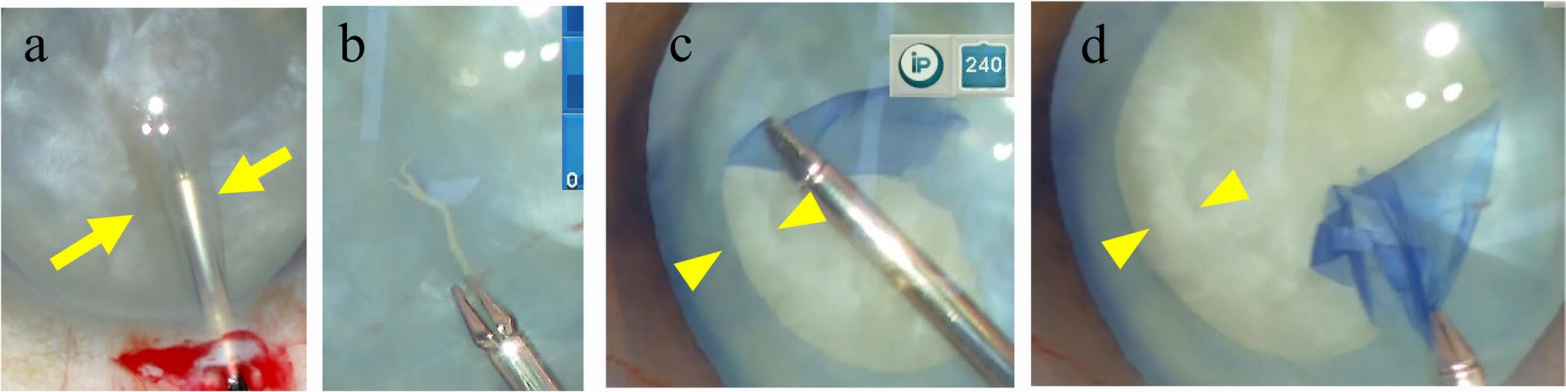
Cortex liquefication and swelling. **a** Leakage of the liquefied cortex (arrow) after puncturing the anterior capsule with forceps. **b–d** After the puncture of the anterior capsule, leakage of the liquefied cortex was not Observed (**b**). However, the cortex near the capsular margin (arrowheads) at the start of the capsulotomy (**c**) moved to the center at the end of the capsulotomy (**d**), indicating that the hydrated swollen cortex had bulged out.

Liquefication and swelling of the cortex cause elevation of ILP [2, 9]. We used surgical videos to determine whether the liquefied cortex had leaked or been aspirated, or whether the hydrated swollen cortex had bulged (Fig. 2). Two judges examined whether these findings were observed. If both judges confirmed these findings, the cataract was classified as an EWC. If no such findings were observed, the cataract was classified as an NWC.

### Image evaluation

**Fig. 3:**
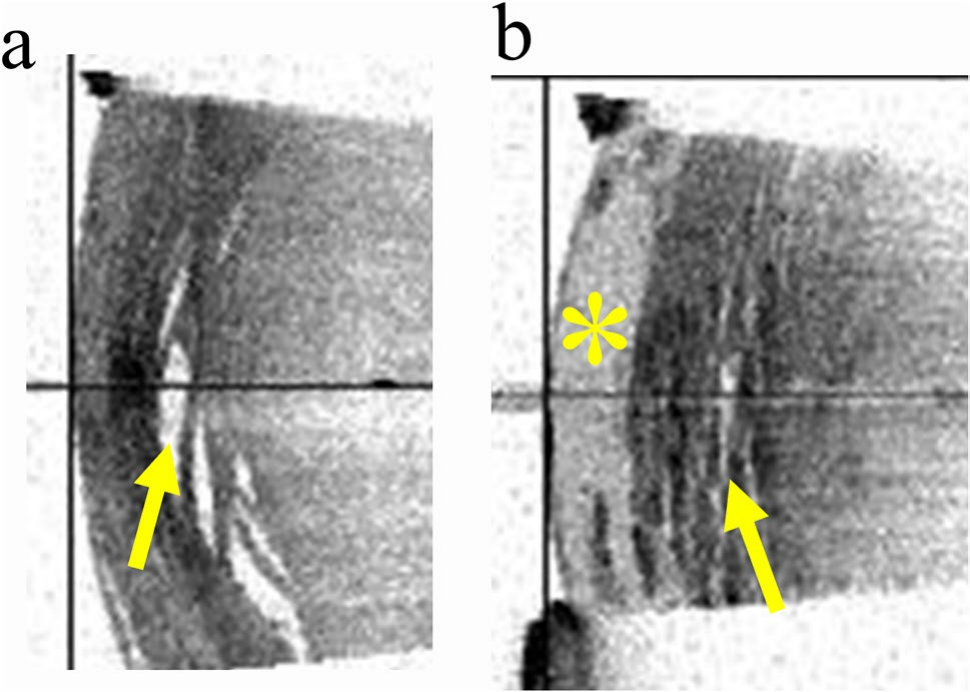
SS-OCT images of the anterior portion of the lens. **a** Intralenticular clefts (arrow) were observed in the cortex near the border of the nucleus. **b** In addition to intralenticular clefts (arrow), a homogenous ground-glass appearance (*) was observed in the cortex under the anterior capsule.

Previous studies using modified AS-OCT and intraoperative OCT show some characteristic appearances in the images of EWC: intralenticular clefts signifying swollen disorganized fibers with localized liquefied cortical material, and/or a homogenous ground-glass appearance signifying confluent cortical liquefaction [2, 17]. Two judges examined whether these features were present in the SS-OCT images (Fig. 3). If both judges determined that the features were present, the image was considered to have the appearance.

### Surgical procedure

All surgery was performed by the two experienced surgeons (D.H. and J.K.). After a superior corneoscleral incision was made using a 2.2- or 2.4-mm steel keratome blade (MANI, Inc.), an ophthalmic viscosurgical device (OVD; DiscoVisc, Alcon Laboratories, Inc.) was injected into the anterior chamber. The anterior lens capsule was stained with trypan blue 0.06% (Sigma-Aldrich Corporation) in all cases, and the OVD was reinjected into the anterior chamber. When a liquefied cortex was observed under the anterior capsule, a 30G needle was inserted into the capsular bag through the center of the anterior capsule, and liquid aspiration was performed. Alternatively, a puncture of the central anterior capsule by forceps without aspiration was performed, and the leaked material was aspirated using a 27G needle if leakage occurred. After CCC, the nucleus and cortex were removed using a CENTURION Vision System (Alcon Laboratories, Inc.), and an acrylic IOL was inserted.

### Statistical analysis

Continuous variables are presented as mean ± standard deviation (SD). To test the difference between two groups, an F-test was performed to verify the quality of the variance, followed by an unpaired t-test. To test the differences between multiple groups, homogeneity of variances was evaluated using Levene’s test. Normally distributed continuous variables were compared using one-way analysis of variance followed by post hoc comparison using Fisher’s least significant difference test. On the other hand, non-normal distributed continuous variables were compared using the Kruskal–Wallis test followed by post hoc comparison using Steel-Dwass test. Categorical variables are presented as absolute and relative frequencies (%) and were analyzed using the chi-square or Fisher’s exact test to assess the incidence rates. Receiver operating characteristic (ROC) curve analysis was used to analyze the ability of the parameters to detect EWC. The Youden index was used to determine the optimal cutoff points. Statistical significance was set at p < 0.05. Data were processed using statistical analysis software (Bell Curve for Excel; Social Survey Research Information Co. Ltd.).

## Results

### Patient characteristics

Forty-eight patients met the inclusion criteria, and their medical records were analyzed. The baseline characteristics of the patients are summarized in Table 1. Of the 48 eyes, a liquefied cortex was observed in 20 eyes in the surgical videos. Among the other 28 eyes, bulging of the swollen cortex was observed in six. These findings were not observed in 22 patients. The 26 eyes with these observations were classified as EWC and the 22 without were classified as NWC. The clinical characteristics of the two groups are summarized in Table 1. No significant differences were observed between the two groups regarding sex or ocular comorbidities. However, the patients with an NWC were older than those with an EWC (Table 1, p = 0.0228, t-test).

**Table 1:** Patient characteristics

Variables	All patients	NWC	EWC	*p* value
	(n=48)	(n=22)	(n=26)	
Age, years	68.8 ± 14.5	73.9 ± 11.8	64.5 ± 15.3	0.0228*
Sex, male	21	8	13	0.3427^a^
Ocular comorbidities				
glaucoma	6	4	2	0.3923^b^
Uveitis	5	1	4	0.3569^b^

^*^t-test, ^a^χ^2^-test, ^b^Fisher’s exact test

### Validity of IOLMaster data

Of the 48 eyes with a WC and fellow eyes, the appropriate LT was measured using the IOLMaster in 22 eyes (45.8%) and 48 eyes (100.0%: p < 0.001, Fisher’s exact test), respectively. For the remaining 26 eyes where a WC could not be measured appropriately, remeasurement using SS-OCT images was required. In the eyes of remeasurement, the anterior surface of the lens was correctly recognized in all eyes. However, the line indicating the posterior surface of the lens was shifted towards the nucleus, and the LT displayed by the IOLMaster was smaller than the true value.

ACDs were appropriately measured using the IOLMaster in all eyes with a WC and in the fellow eyes. ALs were also appropriately measured in all the fellow eyes. However, ALs in eyes with a WC were appropriately measured in only eight eyes (16.7%). In the other eyes with a WC, IOLMaster showed a failed display and we could not determine the fundus edge in the image of SS-OCT. Therefore, the AL data using ultrasound were used for these eyes.

### Parameters (Table 2)

The LT and CT were significantly greater in the EWC eyes than in the NWC eyes or fellow eyes (p < 0.001, ANOVA). The LT and CT in eyes with an NWC were almost the same as those in the fellow eyes. There were no differences in NT between eyes with an EWC, NWC, or fellow eyes (p = 0.6501, ANOVA). The NT ratios were also significantly smaller in EWC eyes than in NWC eyes or fellow eyes (p < 0.001, ANOVA). The difference in LT (ΔLTs) between eyes with an EWC and their fellow eyes was significantly larger than those with an NWC and their fellow eyes (p < 0.001, t-test).

**Table 2:** Comparison of various parameters among eyes with NWC, EWC, and each fellow eyes.

	NWC		EWC		*p* value
	WC	Fellow eyes	WC	Fellow eyes	
LT (mm)	4.28 ± 0.52	4.22 ± 0.31	5.76 ± 0.44*	4.28 ± 0.44	< 0.001^a^
ΔLT (mm)	0.06 ± 0.14		1.48 ± 0.24		< 0.001^b^
NT (mm)	3.12 ± 0.30	3.17 ± 0.28	3.19 ± 0.40	3.08 ±0.34	0.6501^a^
NT ratio (%)	73.2 ± 5.2	75.1 ±4.8	55.6 ± 7.4*	72.4 ± 7.6	< 0.001^a^
CT (mm)	1.16 ± 0.33	1.05 ± 0.23	2.56 ± 0.51^c^	1.20 ± 0.40	< 0.001^d^
ACD (mm)	3.15 ± 0.40	3.19 ± 0.34	2.54 ± 0.42*	3.29 ± 0.41	< 0.001^a^
ΔACD (mm)	-0.04 ± 0.05		-0.75 ± 0.06		< 0.001^b^
AL (mm)	23.24 ± 0.94	23.16 ± 0.95	24.08 ± 1.52^e^	24.02 ± 1.62^e^	0.0224^a^

^*^vs. other three groups, p<0.0001, Fisher’s least significant difference test, ^a^ANOVA, ^b^*t*-test, ^c^vs. other three groups, p<0.0001, Steel-Dwass test, ^d^Kruskal–Wallis test, ^e^vs. eyes with EWC or its fellow eyes, p<0.05, Fisher’s least significant difference,

NWC, normal intralenticular pressure white cataract; EWC, elevated intralenticular pressure white cataract; WC, white cataract; LT, lens thickness; ΔLT, the difference in LT between the eyes with WC and the fellow eyes; NT, nucleus thickness; NT ratio, the ratio of NT to LT; CT, cortical thickness; ACD, anterior chamber depth; ΔACD, the difference in ACD between the eyes with WC and the fellow eyes; AL, axial length.

The ACDs were significantly shorter in EWC eyes than in NWC eyes or fellow eyes (p<0.001, ANOVA). The difference in ACD (ΔACD) between eyes with an EWC and their fellow eyes was significantly larger than the difference between those with an NWC and their fellow eyes (p<0.001, t-test).

There was a significant difference in ALs between eyes with an EWC and an NWC and between their fellow eyes (p=0.0095, ANOVA). However, there was no difference in the ALs between eyes with a WC and fellow eyes for either EWC or NWC.

### Detection ability of parameters for EWC (Table 3, Fig. 4)

ROC curve analysis evaluated the detection ability of LT, ΔLT, NT ratio, CT, ACD, ΔACD, AL, and age for EWC with the area under the ROC curve (AUC) values. The AUC values of these parameters except for ACD, AL, and age, were > 0.970, suggesting that these parameters were effective discriminators. Among them, the detection ability of LT (an AUC of 0.977) for EWC was inferior to those of ΔLT (an AUC of 0.991) and CT (an AUC of 0.984) but was almost the same as ΔACD (an AUC of 0.978). The cutoff values of LT, ΔLT, CT, NT ratio, and ΔACD were 5.12 mm, 0.68 mm, 2.05 mm, 61.8%, and -0.30 mm, respectively.

**Table 3:** The receiver operating characteristic (ROC) curve analysis of various parameters

Parameter	AUC	*P* Value	95% CI	Cutoff Value	Sensitivity (%)	Specificity (%)
LT	0.977	<0.001	0.944-1.010	5.12 mm	96.2	90.9
ΔLT	0.991	<0.001	0.973-1.010	0.68 mm	100.0	95.4
CT	0.984	<0.001	0.959-1.009	2.05 mm	88.5	100.0
NT ratio	0.972	<0.001	0.930-1.014	61.8 %	88.5	100.0
ACD	0.857	<0.001	0.749-0.964	2.73 mm	78.6	80.0
ΔACD	0.978	<0.001	0.948-1.009	-0.30 mm	100.0	86.4
AL	0.681	0.0210	0.527-0.834	23.78 mm	57.7	77.3
Age	0.690	0.0151	0.537-0.843	70.0 yrs	65.4	72.7

AUC, area under the ROC curve; LT, lens thickness; ΔLT, the difference in LT between the eyes with WC and the fellow eyes; CT, cortical thickness; NT ratio, the ratio of NT to LT; ACD, anterior chamber depth; ΔACD, the difference in ACD between the eyes with WC and the fellow eyes; AL, axial length.

**Fig. 4.**
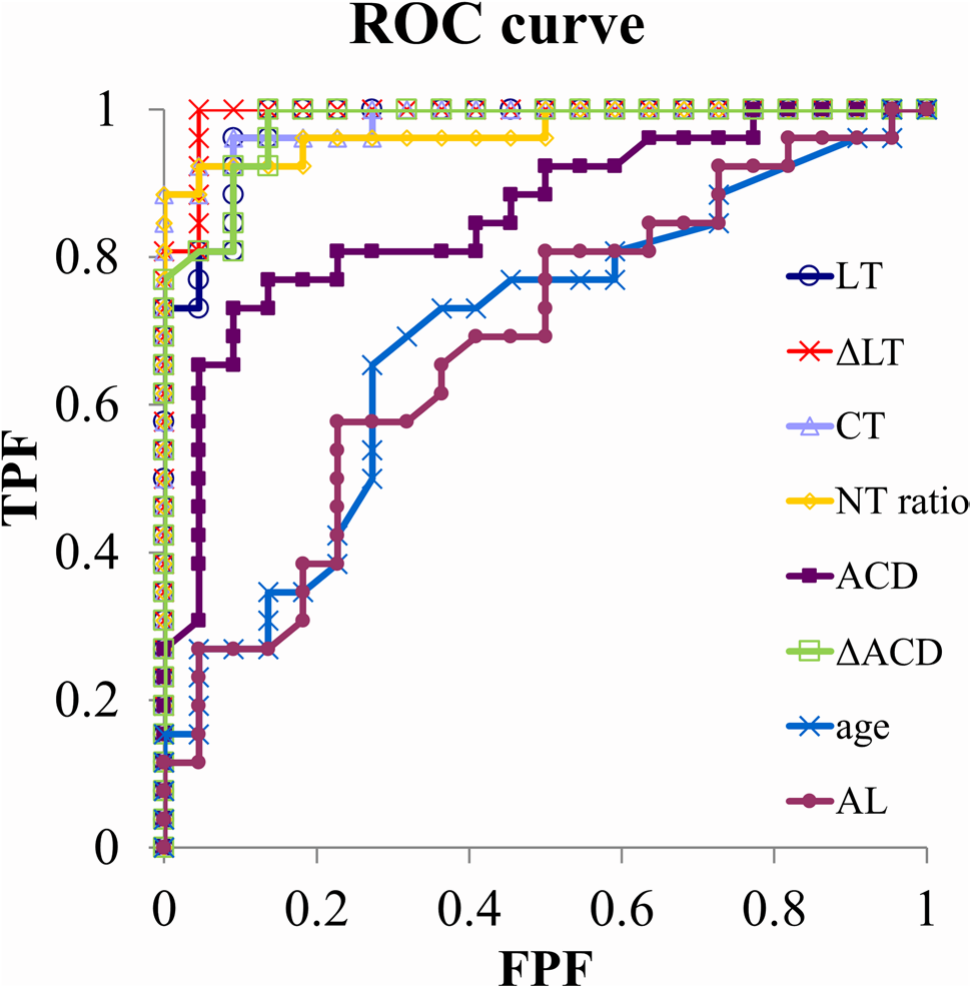
The ROC curves of LT, ΔLT, CT, NT ratio, ACD, ΔACD, AL, and age. LT, lens thickness; ΔLT, the difference in LT between the eyes with WC and the fellow eyes; NT, nucleus thickness; NT ratio, the ratio of NT to LT; CT, cortical thickness; ACD, anterior chamber depth; ΔACD, the difference in ACD between the eyes with WC and the fellow eyes; AL, axial length.

### Image analysis

In SS-OCT images, intralenticular clefts, homogenous ground-glass appearance, and any of them were recognized more frequently in eyes with EWC (18/26, 69.2%, 19/26, 73.1%, 23/26, 88.5%, respectively) than in those with NWC (2/22, 9.1%, 0/22, 0.0%, 2/26, 9,1%, respectively; p < 0.001, Fisher’s exact test). Image evaluation to detect EWC in the presence of intralenticular clefts, homogenous ground-glass appearance, and any of them showed a sensitivity of 69.2%, 73.1%, 88.5%, and a specificity of 90.9%, 100.0%, 90.9%, respectively.

### Surgical outcomes

CCC was achieved in all cases. In one eye with an EWC, part of the CCC unintentionally extended peripherally. Weakness of the zonule was observed in three eyes (two eyes with an EWC and one eye with an NWC), and a capsular tension ring (HOYA) was inserted. Vitreous strand herniation from the zonule during I/A occurred in one eye with an EWC and was cut using scissors. In the present case, the IOL was inserted into the capsular bag because the lens capsule was intact. There were no other serious complications such as posterior capsule rupture. In all cases, the preoperative visual acuity (LogMar = 1.87 ± 0.06) improved after surgery (visual acuity LogMar = 0.13 ± 0.09, p < 0.001, t-test).

## Discussion

This study shows significant differences in LT, ΔLT, CT, NT ratio, ACD, ΔACD, AL, and age between eyes with EWC and NWC. ROC analysis showed that the AUC values of LT, ΔLT, CT, NT ratio, and ΔACD were higher than 0.970, suggesting that these are useful parameters for distinguishing between the two groups. However, the IOLMaster could accurately measure LT in only 45.8% of eyes with a WC, and remeasurement was required in the remaining eyes with a WC. In contrast, it could accurately measure ACD in all eyes with a WC. Because ΔACD was a value that could be obtained by simple subtraction, it was also thought to be a useful parameter that distinguished between NWC and EWC in the IOLMaster. On the other hand, image evaluation by the presence of any of them was useful because it had a sensitivity of 88.5% and a specificity of 90.9%. These findings suggest that the IOLMaster is useful for distinguishing between NWC and EWC.

In this study, the AUC value for LT was greater than for either ACD or age; therefore, LT was a more useful parameter for distinguishing EWC from NWC than ACD or age. This finding is consistent with those of the previous studies [14, 15]. Moreover, Zhang et al. examined the parameters that affected the success rate of femtosecond laser-assisted capsulotomy in WC using A-scan ultrasonography and report that the cutoff point of LT for the success of capsulotomy was 5.21 mm, similar to our cutoff point of LT (5.12mm). In contrast, the AUC value for ΔACD (0.978) was almost the same as that of LT (0.977). ACD is affected by various factors, including age, gender, AL, and LT [21, 23, 27]. Since these factors differ between individuals, ACD may not directly reflect the difference in LT. However, there is symmetry between the left and right eyes of the same person for various factors, such as age, gender, and AL [22, 24, 25]. Therefore, ΔACD, which compared the difference between the left and right eyes of the same person, might reduce the differences in these factors other than LT and more clearly show the differences in LT. For the same reason, the AUC of ΔLT (0.991) might be larger than that of LT (0.977).

The SS-OCT image analysis performed in this study investigated the presence of intralenticular clefts and a homogenous ground-glass appearance in the cortex. Both appearances indicate the presence of liquefied cortical material [2, 17]. Image evaluation by a homogenous ground-glass appearance had a high specificity (100.0%), which may not lead to misjudgment of EWC. However, its sensitivity (73.1%) was not so high, which implies that this sign did not sufficiently capture EWC. Image evaluation by the presence of intralenticular clefts had lower sensitivity (69.2%) and specificity (90.9%). These image evaluation results may be due to differences in the appearance stage. From the early stages of cortical liquefaction, intralenticular clefts were observed, and as liquefaction progressed, a homogenous ground-glass appearance was visible [2, 17]. A homogenous ground-glass appearance may not be observed in the early stages of EWC. Intralenticular clefts may be recognized before the ILP is sufficiently raised.

Using a slit-lamp biomicroscope [3], modified OCT [17], and intraoperative OCT [2], only the anterior portion of the lens can be examined. However, the SS-OCT images of this study showed a lens image from the anterior surface to the posterior surface, which resulted in an evaluation of the nucleus size. Although the LT, CT, and NT ratios of EWC were significantly different from those of NWC and fellow eyes (p < 0.0001, ANOVA), the NT of EWC was similar to that of NWC and the fellow eyes (p < 0.6501, ANOVA). This finding suggests that the increase in the LT of EWC was due to the increase in CT: liquefaction of the cortex, consistent with previous reports [1, 17]. Moreover, the CT increased only in the eyes with EWC, resulting in an increase in LT only in the eyes with EWC. Therefore, LT was thicker and ACD was shallower in the eyes with EWC than in the fellow eyes, but not in the eyes with NWC. This suggests that ΔLT and ΔACD are useful for detecting EWC.

In this study, the AL was longer in the eyes with EWC than with NWC. The same difference was observed in their fellow eyes. There was no difference in AL between the eyes with WC and the fellow eyes in both EWC and NWC. The cause of this difference is unknown, but it was thought that the difference in WC status was not the cause of the difference in AL.

This study had some limitations. Firstly, the single-center and retrospective design restricts the generalizability of the findings. Future multicenter studies should confirm the usefulness of the cutoff values and diagnostic performance of the biometric and imaging parameters. Secondly, this study does not include Morgagnian-type WC, in which the cortex is completely liquefied and the nucleus usually sinks into the inferior capsular bag by gravity. However, Morgagnian-type WC may be easily detected because the SS-OCT image will show the sinking of the nucleus. Thirdly, ΔACD and ΔLT were invalid when the fellow eye was with a WC or pseudophakic. In such cases, evaluating only the eye with a WC is necessary. When LT is measured appropriately using the IOLMaster, the EWC can be detected from the LT. However, LT can only be measured correctly in 45.8% of eyes with a WC. Without an accurate LT, it is necessary to evaluate using only image analysis or to remeasure SS-OCT images. The easiest method to remeasure the parameters is the NT ratio, which can be obtained from the relative distance of LT and NT without actual data.

In conclusion, the LT, ΔLT, ΔACD, and SS-OCT images obtained with the IOLMaster 700 were useful for detecting EWCs. Although the LT could be accurately measured in only 45.8% of eyes with a WC, the ΔACD could be accurately measured in all cases and had almost the same detection ability as the LT. These findings may suggest that the IOLMaster 700 is useful for detecting EWC.
